# Computer analysis shows differences between mitochondrial miRNAs and other miRNAs

**DOI:** 10.18699/vjgb-24-91

**Published:** 2024-12

**Authors:** P.S. Vorozheykin, I.I. Titov

**Affiliations:** Novosibirsk State University, Novosibirsk, Russia; Novosibirsk State University, Novosibirsk, Russia Institute of Cytology and Genetics of the Siberian Branch of the Russian Academy of Sciences, Novosibirsk, Russia Kurchatov Genomic Center of ICG SB RAS, Novosibirsk, Russia

**Keywords:** mitomiR, mitochondria, miRNA, evolution, database, митомиР, митохондрия, микроРНК, эволюция, база данных

## Abstract

A subclass of miRNAs with as yet unknown specific functions is mitomiRs – mitochondrial miRNAs that are mainly derived from nuclear DNA and are imported into mitochondria; moreover, changes in the expression levels of mitomiRs are associated with some diseases. To identify the most pronounced characteristics of mitochondrial miRNAs that distinguish them from other miRNAs, we classified mitomiR sequences using the Random Forest algorithm. The analysis revealed, for the first time, a significant difference between mitomiRs and other microRNAs by the following criteria (in descending order of importance in the classification): mitomiRs are evolutionarily older (have a lower phylostratigraphic age index, PAI); have more targets and disease associations, including mitochondrial ones (two-sided Fisher’s exact test, average p-values 1.82 × 10–89/1.13 × 10–96 for all mRNA/diseases and 6.01 × 10–22/1.09 × 10–9 for mitochondria, respectively); and are in the class of “circulating” miRNAs (average p- value 1.20 × 10–56). The identified differences between mitomiRs and other miRNAs may help uncover the mode of miRNA delivery into mitochondria, indicate the evolutionary conservation and importance of mitomiRs in the regulation of mitochondrial function and metabolism, and generally show that mitomiRs are not randomly encountered miRNAs. Information on 1,312 experimentally validated mitomiR sequences for three organisms (Homo sapiens, Mus musculus and Rattus norvegicus) is collected in the mitomiRdb database (https://mitomiRdb.org).Key words: mitomiR; mitochondria; miRNA; evolution; database.

## Abstract (ru)

Одним из подклассов микроРНК с до сих пор неизвестными специальными функциями являются митомиРы (mitomiRs) – митохондриальные микроРНК, которые в основном происходят из ядерной ДНК и импортируются в митохондрии, при этом изменение уровня их экспрессии ассоциировано с рядом заболеваний. Для выявления характерных особенностей митохондриальных микроРНК, отличающих их от остальных микроРНК, мы провели классификацию этих последовательностей с помощью метода случайного леса. Проведенный анализ впервые выявил достоверные различия между митомиРами и микроРНК по следующим характеристикам (по убыванию степени их важности в классификации): митомиРы имеют достоверно больший эволюционный возраст (низкий индекс филостратиграфического возраста, PAI), большее количество мишеней и ассоциаций с болезнями, в том числе митохондриальными (двусторонний точный тест Фишера, средние p-значения 1.82 × 10–89/1.13 × 10–96 для всех мРНК/болезней и 6.01 × 10–22/1.09 × 10–9 для митохондриальных); принадлежат к классу «циркулирующих» (среднее p-значение 1.20 × 10–56). Обнаруженные различия между митомиРами и остальными микроРНК могут помочь раскрыть способ доставки микроРНК в митохондрии, свидетельствуют об эволюционной консервативности и важности митомиРов в регулировании функций и метаболизма митохондрий, а в целом говорят о том, что митомиРы не являются случайными микроРНК. Информация о 1312 экспериментально подтвержденных последовательностях митомиРов для трех организмов (Homo sapiens, Mus musculus и Rattus norvegicus) собрана в базе mitomiRdb (https://mitomiRdb.org).

## Introduction

Mitochondria engage in extensive bidirectional inter-compartmental crosstalk to regulate their proteome, overall cellular fitness and organismal health. To date, it is well known that the fundamental pathways of the miRNA biogenesis start in the nucleus and end in the cytoplasm (Bartel, 2018; Salim et al., 2022; Ziętara et al., 2023). However, there is evidence that these short non-coding RNA sequences are also present in organelles, in particular, in mitochondria (Lung et al., 2006; Kren et al., 2009). In many cases, mitochondrial microRNAs (the so-called mitomiRs) are more abundant in the mitochondria than in the cytoplasm. These observations suggest a nucleus miRNA translocation into mitochondria and/or the existence of a complete miRNA maturation process within mitochondria.

The existence of a transport mechanism is supported by the detection of the so-called circulating miRNAs (Pozniak et al., 2022). There are also arguments in favor of the second option: first, the miRNA machinery proteins AGO2 and Dicer, which are involved in the canonical pathway of microRNA biogenesis, have been found in mitochondria (Bandiera et al., 2011; Wang W.-X. et al., 2015); second, mitochondrial gene expression can be regulated by mitochondrial miRNAs and this regulation inevitably manifests itself in mitochondriarelated diseases (Li et al., 2012; Tomasetti et al., 2014; Zhang et al., 2014; Lin, Chu, 2021; Erturk et al., 2022; Gohel, Singh, 2022). Since the composition of miRISC (miRNA-induced silencing complex) varies at different development stages, this suggests the possibility of a mitochondria-specific miRNA origin and biogenesis, as well as potentially unknown functions of nuclear miRNAs within mitochondria. This highlights mitochondrial miRNAs as a new subclass of miRNAs with significant implications for scientific research. Nevertheless, the specific functions and biogenesis pathways of mitomiRs remain unexplored, and it is still unclear whether mitomiRs are merely typical microRNAs that happen to be observed in mitochondria by chance.

To reveal specific features of this new miRNA class, we analyzed all miRNA sequences using the Random Forest algorithm and determined the most important criteria for miRNA classification (listed in descending order of their importance): the phylostratigraphic age index (PAI) of the miRNA; the presence of miRNA targets, and whether the miRNA belongs to the “circulating” class of miRNAs. Based on the obtained data, we drew conclusions regarding the age of mitomiRs, their possible appearance in mitochondria, and their significance for the organism functioning.

The explored mitomiRs have been collected in the mitomiRdb database (https://mitomiRdb.org) – a manually curated repository of experimentally discovered mitochondrial miRNAs. This database stores information about mitomiRs for three mammals: Homo sapiens, Mus musculus and Rattus norvegicus. There are 1,312 annotated sequences with details such as identifiers, nucleotide sequences, and secondary structures of precursors. Additionally, the database provides references to publications with supporting experiments and evidences of experimentally validated miRNA-mRNA and miRNA-disease associations, including those related to mitochondria. All collected data are available online and can be freely downloaded for further computational analysis.

## Materials and methods

Mature miRNA sequences were downloaded from the miRBase database (https://miRBase.org, releases 10–22.1) (Kozomara et al., 2019). The latest release of the database contains 48,885 annotated miRNA sequences from 285 species. The total number of H. sapiens, M. musculus and R. norvegicus miRNAs – 5,398 sequences, of which 2,274 were marked as “high confidence” by database curators (those miRNAs, the reads of which align with the canonical pre-miRNA processing patterns by Drosha/Dicer complexes).

To study the relationship of mitomiRs with mRNA, we used the miRTarBase database (https://mirtarbase.cuhk.edu.cn, release 8.0) (Huang et al., 2020) – a manually curated repository of experimentally validated microRNA-target interactions from scientific publications with experimental evidence of direct interactions. The total number of annotated entries of microRNA-mRNA interactions for human, mouse and rat miRNAs is equal to 553,118. Among these, 13,311 entries are noted as “supported by strong experimental evidence”, while the remaining 539,807 entries are based on “weak” proof.

Data on experimentally validated microRNA-disease associations were obtained from the RNADisease database (http:// www.rnadisease.org, release 4.0, “Experimental data” section, miRNA-disease information entries) (Chen et al., 2022). Each association was manually curated from publications, with particular attention being paid to experimental evidence of the miRNA role in regulation and pathogenesis of diseases as well as the analysis of miRNA-mRNA complementary binding and its involvement in disease progression. The total number of annotated entries for the three considered species (human, mouse and rat) amounts to 211,150.

The following mtDNA reference sequences were used to determine the localization of mitochondrial miRNAs and the mitochondrial genes: H. sapiens (NC_012920.1), M. musculus (NC_005089.1), and R. norvegicus (NC_001665.2) (Sayers et al., 2022). To explore the evolution of the let-7a-5p binding site, the following mitochondrial genomes of primates were used: Gorilla gorilla (NC_001645.1), Pan paniscus (NC_001644.1), Pongo pygmaues (NC_001646.1), Pan troglodytes (NC_001643.1), and Symphalangus syndactylus (NC_014047.1) (Sayers et al., 2022).

To calculate the phylogenetic age index (PAI) of miRNAs, we took the taxonomic lineages from the NCBI server (https:// ftp.ncbi.nlm.nih.gov/pub/taxonomy/new_taxdump, data as of July 12, 2022) (Sayers et al., 2022). For each miRNA sequence from 285 organisms, all its homologous sequences were identified to determine the distribution of similar microRNAs across the species. Two nucleotide sequences were considered homologous (phylogenetically related) if the Hamming distance of their globally aligned sequences was less than 10 % of the alignment length. Alignment parameters: a match score of 5.0, a mismatch penalty of –4.0, an initial insertion/deletion penalty of –10.0, and an extending insertion/deletion penalty of –0.5. To calculate the PAI of the miRNA sequence, we used a set of organisms in which miRNA homologues appeared. According to taxonomic lineages, the PAI value represents the serial number of the most common taxon of this set (numbering from zero) (Mustafin et al., 2019).

DO- and KEGG-identifiers and names of diseases were obtained from the Disease Ontology Project (Schriml et al., 2022) and the Kyoto Encyclopedia of Genes and Genomes (Kanehisa et al., 2017). With that information, we have compiled a list of mitochondria-associated diseases. For this purpose, we took names and identifiers of diseases that are included in the supergroup (and its subgroups) KEGG H01427 (Mitochondrial diseases) and those explicitly mentioning mitochondria in their names. Furthermore, a list of mitochondria-associated diseases with the total number of 74 KEGG and 185 DO ID entries was made (data provided in Supplementary Material 1)1.


Supplementary Materials are available in the online version of the paper: https://vavilovj-icg.ru/download/pict-2024-28/appx27.xlsx


Information on “circulating” miRNAs was retrieved from the miRandola database (release on February 2017, 606 miRNAs) and the plasmiR database (release on June 17, 2021, 251 miRNAs) (Russo et al., 2018; Tastsoglou et al., 2021). These extracellular miRNAs are detected in traceable quantities in blood and other body fluids. The total number of circulating miRNAs from two databases is 628 (590 human, 18 mouse, and 20 rat miRNAs).

**Fig. 1.**
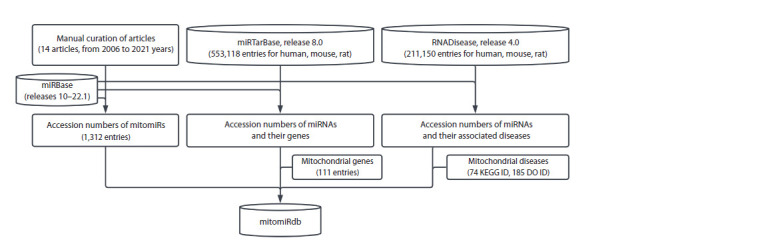
A schematic workflow for collecting information about mitomiRs to form the mitomiRdb database.

Figure 1 presents a schematic workflow outlining the process of gathering information about mitomiRs and establishing connections between mitomiRs and their targets or associated diseases. First, we selected the papers, which explore mitochondria-located miRNAs or contain references to the term “mitomiR”. Out of them, we took 14 articles (published between 2006 and 2021) that reported the experimentally verified presence of miRNA sequences within mitochondria isolated from three mammal species (H. sapiens, M. musculus, R. norvegicus) for different cell types and tissues (Lung et al., 2006; Kren et al., 2009; Bian et al., 2010; Bandiera et al., 2011; Barrey et al., 2011; Mercer et al., 2011; Das et al., 2012; Sripada et al., 2012; Dasgupta et al., 2015; Jagannathan et al., 2015; Wang W.-X. et al., 2015; Wang X. et al., 2017; Fan et al., 2019; Zheng et al., 2021). In these studies, miRNAs are mentioned by their names (e. g., hsa-miR-1), which may have changed over time in the miRBase database. To ensure consistency, based on the miRBase annotation history, we matched each miRNA name with its corresponding unique accession number (MIMAT number) from the miRBase. The accession number allows the unambiguous identification of a mitomiR sequence across database releases.

During the matching process, we found that some previously annotated mitomiRs had been excluded from the recent miRBase releases. To ensure comprehensive coverage, we extended our dataset of mitomiRs to include 40 additional miRNAs and their 41 precursors that had been previously annotated in the miRBase database (Supplementary Material 2). As a result, we compiled accession numbers, sequences, secondary structures of precursors, and other additional information for 1,312 mitomiRs that were enriched in mito-chondria.

To characterize mitochondria-associated miRNAs, in addition to the mitomiRs sample, we compiled a dataset of 4,126 sequences (referred to as non-mitomiRs), which contains human, mouse and rat miRNAs from the miRBase database (release 22.1) except all identified mitomiRs.

Then, using the naming history of the miRBase sequences, for each miRTarBase and RNADisease entry, its miRNA identifier (ID) was matched to the unique miRBase accession number for further possibility of unambiguous association of microRNAs with targeted genes and related diseases. For some entries it was not possible to clearly identify an accession number of miRNA due to incomplete or inconsistent database information (e. g., hsa-miR-b5539 and hsa-miRPlus-C1100 are not identifiable miRBase IDs; the database entry contains pre-miRNA name hsa-let-7a-1, which cannot be unequivocally matched to a single miRNA in the miRNA-miRNA duplex). By removing these ambiguous entries, we established reliable references between identified mitomiRs and their respective targets and diseases.

Finally, having the set of mitomiR-target and mitomiRdisease associations, we compared how mitomiRs are related to known mitochondrial genes and diseases. We have included in the database the information on relationships between mitomiRs and 111 known mitochondrial genes (which encode for rRNAs, tRNAs, and protein subunits) for three (human, mouse, rat) examined mtDNAs. Each entry in the RNADisease database provides the name of disease and one or several disease identifiers: Disease Ontology (DO) ID (Schriml et al., 2022), MeSH ID (Sayers et al., 2022), and KEGG ID (Kanehisa et al., 2017; Schriml et al., 2022). Therefore, for each mitomiR sequence, we additionally indicated its connection (or a lack of connection) to the prepared list of mitochondrial diseases (Supplementary Material 3) as well as to all diseases.

To identify and rank the most powerful characteristics of mitomiRs in comparison to other miRNAs (non-mitomiRs), we analyzed miRNA sequences using the Random Forest algorithm (Breiman, 2001). Four binary and one numerical criteria were chosen for classification. Binary criteria: (1) whether the miRNA sequence is “circulating”; (2) whether the miRNA is “confident” according to the miRBase declaration; (3) whether the miRNA has a validated target; and (4) whether the miRNA is associated with a disease. The numerical criterion was the PAI value of the miRNA sequence.

The Random Forest algorithm was carried out 100 times on specific datasets: each dataset consists of all mitomiRs and non-mitomiRs except all homologous sequences but one (randomly selected) member from each homologue group. In each iteration, a randomly generated subset (one third of the total dataset) serves as a test dataset, while the remaining part is used for model training. Statistical estimates and significance levels for the criteria were averaged over all tests.

## Results

Statistics. Considering the papers with the data on mitochondria- located miRNAs for three mammal species (Homo sapiens, Mus musculus, and Rattus norvegicus), we obtained information on 1,312 accession numbers of the mitomiR sequences. Among them, there were sequences that had been excluded from the miRBase database for various reasons. For example, miRNAs hsa-miR-1974, hsa-miR-1977 and hsa-miR-1978 overlap with mitochondrial tRNAs; hsa-miR- 6723-5p has a reads pattern from RNA-seq experiments that does not support its annotation as a miRNA in the miRBase; mmu-miR-2145 is a fragment of 5S rRNA; and other entries are suspected of being transcriptional noise or products of non-canonical maturation process. Approximately 66.6 % (874) of the discovered mitomiRs correspond to human miRNAs, while the rest belong to mouse (30.6 %, 401) and rat (2.8 %, 37).

By comparing mitomiR sequences and names, we found 16 (out of possible 37, based on the number of mitomiRs in R. norvegicus) conserved mitomiRs, i. e. those detected in mitochondria across all of the three considered species. Additionally, 30.6 % of all mitomiRs have been described in more than one publication, which may represent their higher credibility as mitochondrial. Only nine of the mitomiR sequences (hsa-miR-1973, hsa-miR-1974, hsa-miR-1977, hsa-miR-1978, hsa-miR-4461, hsa-miR-4463, hsa-miR-4284, hsa-miR- 4485-3p, mmu-miR-805) are fully mapped to mitochondrial DNA (three to tRNAs and rRNAs, two to protein-coding regions, and one to the D-loop). Notably, among them, only five mitomiRs (hsa-miR-1973, hsa-miR-1974, hsa-miR-1977, hsa-miR-1978, hsa-miR-4485-3p) have been additionally validated by RT-PCR/RT-qPCR/qRT-PCR analysis or observed in mitochondria in greater abundance than in the cytoplasm.

**Fig. 2.**
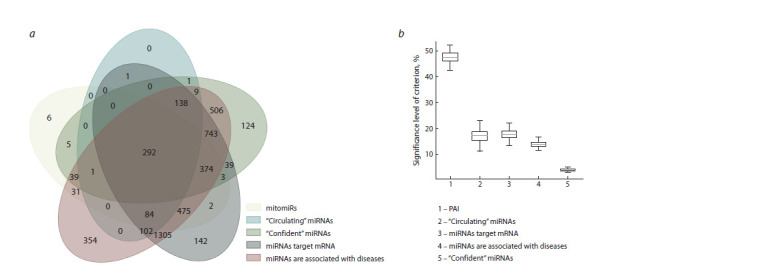
Comparison of microRNA class criteria. а, the Venn diagram illustrates the distribution of miRNA sequences across five classes: mitomiR sequences, “confident” miRNAs, “circulating” miRNAs and the miRNA sequences with known target and disease associations; b, the significance levels of criteria for mitomiR prediction, as determined by the Random Forest algorithm (averaged values over 100 tests). The horizontal line indicates the average significance level, the “whiskers” extend from the box to the farthest data point lying within 1.5 times the inter-quartile range from the box.

Criteria. The selected characteristics of mitomiRs (in comparison with the rest of miRNAs) do not allow classifying mitomiRs by one of the chosen criteria (Fig. 2a). However, the Random Forest classification algorithm ranks criteria by their influence, highlighting the most important characteristics of mitomiRs. With the considered criteria, the Random Forest model achieved average prediction errors (the fraction of incorrectly classified samples) of 0.20 ± 0.003 for the training dataset, and 0.22 ± 0.006, for the test dataset. The most influential criteria for the classification were: (1) the PAI value of the miRNA, (2) the presence of miRNA targets, and (3) whether the miRNA is classified as circulating (Fig. 2b). In contrast, the least important criteria were the miRNA’s association with disease and its confidence level (as defined by miRBase); miRNA confidence plays the minimal role in the classification.

**Fig. 3.**
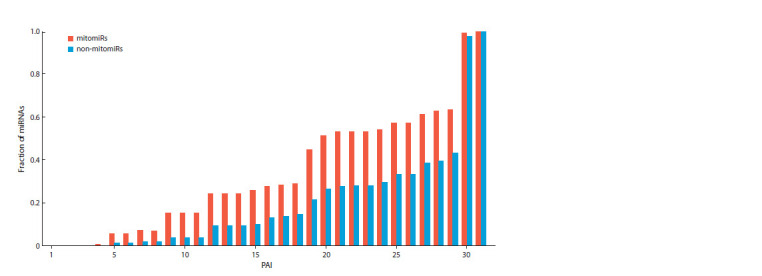
Cumulative distribution of mitomiR and non-mitomiR sequences by PAI values. The fraction of mitomiRs with a PAI value less than 16 exceeds the corresponding fraction of non-mitomiRs, with the significance level of 1.10 × 10–41 ± 5.97 × 10–41. The minimum PAI value is 4, which corresponds to four mitomiRs (hsa-miR-99a-5p, mmu-miR-99a-5p, hsa-miR-100-5p, mmu-miR-100-5p) and two non-mitomiRs (rno-miR-99a-5p, rno-miR-100-5p) from the abundant miRNA family mir-10.

The evolutionary characteristic PAI (phylostratigraphic age index, describes the age of a mitomiR) appeared to be the most significant criterion for mitomiR classification. PAI denotes the serial number of a taxon (node of the phylostratigraphic tree) furthest from its root and occurring in taxonomic lineages of the microRNA sequence and its homologues. According to the PAI values, mitomiR sequences generally show, on average, greater evolutionary conservation than non-mitomiRs. The minimum value of mitomiRs’ PAI is 4 (Fig. 3). Only four mitomiRs (hsa-miR-99a-5p, mmu-miR-99a-5p, hsamiR- 100-5p, and mmu-miR-100-5p) and two non-mitomiRs (rno-miR-99a-5p, rno-miR-100-5p) have this PAI due to their homologs being found in Nematostella vectensis, which testifies to an ancient history of these miRNAs’ origin (Grimson et al., 2008).

However, in the evolutionarily distant species, miRNA sequence homology, even with the presence of a hairpin structure, does not guarantee the existence of a real miRNA (Grimson et al., 2008). All the aforementioned mitomiRs belong to the abundant miRBase family mir-10. This family also contains a set of human and mouse mitomiRs (miR-10a, miR-10b, miR-125b from the 5p-branch of precursors) with the PAI of 5. In contrast, the rat miRNAs from this family correspond to non-mitomiRs, which is probably due to the limited number of mitomiRs found in the rat. It is known that abundant miRNA families, such as mir-10, tend to be older, more efficient, target more genes, and are more likely to be associated with diseases. All of these factors point out the importance of mitomiRs in mitochondrial function and metabolism.

The next important criteria for the mitomiR classification are the presence of miRNA-target associations and whether the miRNA is classified as circulating. The total number of the mitomiR-mRNA interactions is 23,151, which includes 3,318 entries with “strong” evidence of interactions and 19,833 entries with “weak” evidence (Supplementary Material 4). It should be noted that the considered subset of miRTarBase does not contain entries with interspecies interactions, meaning there are no observations where the species of the miRNA does not match the species of the targeted mRNA. Notably, a significantly fewer number of mitomiRs (in contrast to the number of non-mitomiRs) are associated with approximately the same number of mRNAs. Two-sided Fisher’s exact test (average p-value 1.82×10–89 ± 7.73×10–89) demonstrates a significant connection between the type of miRNA (mitomiR or non-mitomiR) and its association with mRNA (see the Table). All of this may indicate the important regulatory role of mitochondrial miRNAs

**Table 1.**
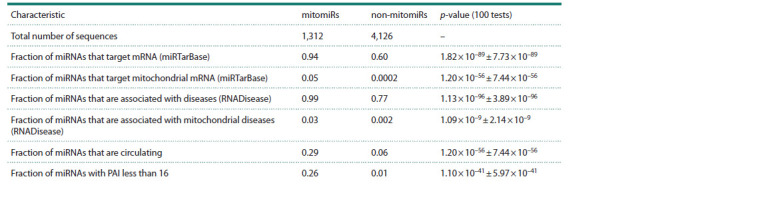
Characteristics, for which significant differences between mitomiRs and other miRNAs are observed Notе. The significance of differences between the characteristics was evaluated by averaging results over 100 iterations, each involving the random selection of one miRNA sequence from each group of homologous.

Further, having a set of mitomiR-mRNA associations, we considered only mitochondrial genes and their connections to mitomiRs. The total number of such genes (which encode for rRNAs, tRNAs, and protein subunits) across the three examined mtDNAs is 111. A notable feature of the miRTarBase database is that it provides information only on protein-coding mitochondrial genes and does not cover RNA-coding genes. Additionally, we found that 65 mitomiRs target mitochondrial mRNA, while 1,247 do not. Moreover, sequences of all targeting mitomiRs are not mapped to the mtDNA, meaning that these mitomiRs are external to mitochondria. The mitomiRs target 12 mitochondrial mRNAs: ND1, ND2, ND3, ND4, ND4L, ND5, ND6, COX1, COX2, COX3, CYTB, ATP6. The largest number of mitomiRs (more than 15) target only two human mRNAs (ATP6 and COX1), while the lowest number of mitomiRs (fewer than 5) is associated with the human mRNAs ND3 and ND4L, as well as the mouse and rat mRNAs COX1. Furthermore, among 4,126 non-mitomiRs, only one (hsa-miR- 15a-3p) targets mitochondrial mRNA (ND4L). Two-sided Fisher’s exact test (average p-value 6.01×10–22 ± 2.30×10–21) demonstrates a significant association between the type of microRNA (mitomiR or non-mitomiR) and its interaction with mitochondrial mRNA.

The significance of the “circulating” miRNA criterion may reflect a specific mode of mitomiR transportation into mitochondria. Comparison between mitomiRs and non-mitomiRs (Fig. 2a) shows that mitomiRs are more prevalent among circulating miRNAs than non-mitomiRs (377 mitomiRs vs 251 non-mitomiRs). Two-sided Fisher’s exact test (carried out on miRNA sets purified from homologous sequences) yielded an average p-value of 1.20×10–56 ± 7.44×10–56.

Although association with diseases demonstrates lower significance for classification than the previously mentioned criteria (due to its similarity with the “presence of targets” criterion, see the Table and Supplementary Material 4), it remains essential for understanding mitomiR functions. For each mitomiR and non-mitomiR entry, we indicated its connection (or a lack of connection) to mitochondrial diseases. Using entries from the RNADisease database, we discovered 36 mitomiRs (out of 1,312) associated with mitochondrial diseases based on their names or identifiers. On the other hand, only 9 out of 4,126 non-mitomiRs had the same association, which may be due to the targeting of nuclear mRNAs producing mitochondria-localized product. Both mitomiRs and non-mitomiRs showed associations with the disease group “Mitochondrial disease” and MNGIE-syndrome (Supplementary Material 3). Two-sided Fisher’s exact test (average p-values 1.13×10–96 ± 3.89×10–96/1.09×10–9 ± 2.14×10–9 for all and for mitochondrial diseases, respectively) confirmed that mitomiRs are more closely related to diseases (including mitochondrial) than non-mitomiRs. The associations with mitochondrial mRNA and diseases suggest an important role of mitomiRs in mitochondria activity.

Data. The mitomiRdb database (https://mitomiRdb.org) offers a web-based user interface for accessing mitomiR data and for performing information extraction. The database includes the following data (according to miRBase): unique identifier (MIMAT), name, nucleotide sequence, and the organism in which the mitochondrial miRNA was observed. In addition, a mitomiR’s confidence flag highlights entries which are associated with mitochondrial mRNA or diseases and those mapped to the mitochondrial genome. The database provides additional information about the secondary structure of miRNA precursors, references to the supporting publications, and the list of associated diseases and genes. For entries classified as “confident” mitomiRs, a list of associated mitochondrial genes and diseases is provided, along with a note indicating the presence of the mitomiR sequence in mtDNA. All the data presented are available for download in SQLite format for further computational analysis (doi.org/10.6084/ m9.figshare.22592380).

## Discussion

To date, numerous microRNAs have been detected in mitochondria. It is still unknown whether the presence of these miRNAs is due to their functional roles or it is simply a coincidence that random miRNAs have been observed in these organelles. If the former is true, mitochondrial miRNAs may have special features of biogenesis and specific regulation of the expression of genes, including mitochondrial ones. In this study, we analyzed the characteristics of miRNAs to identify the factors that distinguish mitomiRs from other microRNAs and to confirm the fact that the observation of this miRNA class is not by chance.

The most significant feature of mitomiRs is the phylostratigraphic age index (PAI), which characterizes the evolutionary age of miRNA sequences. A smaller PAI for mitomiRs indicates that, on average, mitomiRs are older than non-mitomiRs. Like most old miRNAs, they are more frequently involved in a greater number of important regulatory processes, including those related to mitochondrial functions.

A significant association of mitomiRs with mRNAs (including mitochondrial ones) has been revealed based on experimentally determined interactions of microRNAs with targets. This suggests that the presence of mitomiRs within mitochondria is not by chance, and underscores the importance of mitomiRs for the functioning of the entire organism and the mitochondria in particular. Importantly, miRNA-mRNA interactions do not necessarily result in gene silencing. Approximately half of the Argonaute mRNA crosslinks involve miRNA-mRNA bindings that lack a contiguous match to miRNA seed nucleotides (Grosswendt et al., 2014), which are most critical for target association (Chandradoss et al., 2015; Salomon et al., 2015). These non-canonical binding sites, although identified by crosslinking (CLIP-methods), do not always mediate gene expression (Agarwal et al., 2015). Therefore, evolutionary conservation may serve as useful evidence of site functionality. To test this hypothesis for a single mitomiR example, we selected the only site where the crosslinking study aligned with the computer prediction (Khorsandi et al., 2018). This site is responsible for targeting mt-ND5 by miRNA hsa-let-7a, it resides between positions 13,418–13,439 of human mtDNA and in roughly the same position (1,081 bp from the ND5 start) in other primates. However, the site appears in the human due to a synonymous nucleotide substitution (C>T) in the site position that corresponds to the second seed nucleotide of let-7a. Meanwhile, in this position, there is a backward SNP (T>C) (rs386829181, 7×10–4 allele frequency) (Sherry, 2001), which has been associated with cranial meningiomas in Chinese patients.

The next factor that distinguishes mitomiRs from other microRNAs is their assignment as circulating miRNAs. Circulating miRNAs are a type of extracellular RNAs that are observed in sufficient quantities in various body fluids. The importance of this factor for the mitomiR classification suggests a potential similarity between the mechanisms of free miRNA transfer out of the cell and the translocation of mitomiRs within mitochondria.

The criterion based on mitomiR-disease associations appears less important for classification, possibly due to the overlapping associations of both mitomiRs and non-mitomiRs with targets and diseases (Supplementary Material 4). Despite this, the criterion demonstrates a significant connection between diseases and mitomiRs, which may imply that mitomiRs play an important role in regulating various biological processes, including those related to mitochondria.

The least important factor is the “confidence” of miRNA, as defined by miRBase standards. This may indicate the existence of an unknown maturation pathway for mitomiRs, which forms a microRNA-microRNA duplex with non-canonical overhanging ends, rather than the canonical 2-nucleotide overhangs that arise from Dicer and Drosha cleavage of premiRNA.

Despite the presence of mitochondria in all cell types of the studied mammals, the contribution of tissue specificity factor and miRNA expression levels to the difference between mitomiRs and non-mitomiRs cannot yet be assessed. This limitation arises from the fact that existing experimental observations of mitomiRs cover a small number of tissues and provide insufficient information about the expression of mitomiRs.

## Conclusion

The following characteristics of mitochondrial miRNAs allow to separate mitomiRs from other microRNAs (in descending order of importance): phylostratigraphic age index (PAI), the presence of microRNA targets, and the classification of microRNAs as “circulating”. These identified characteristics may help to shed light on the origin, processing and function of mitomiRs.

All experimentally investigated mitomiRs have been collected in the mitomiRdb database (https://mitomiRdb.org). The database may be useful for a more comprehensive study of microRNAs and their subclass of mitomiRs.

## Conflict of interest

The authors declare no conflict of interest.

## References

Agarwal V., Bell G.W., Nam J.-W., Bartel D.P. Predicting effective
microRNA
target sites in mammalian mRNAs. eLife. 2015;4:
e05005. doi 10.7554/eLife.05005

Bandiera S., Rüberg S., Girard M., Cagnard N., Hanein S., Chrétien D.,
Munnich A., Lyonnet S., Henrion-Caude A. Nuclear outsourcing of
RNA interference components to human mitochondria. PLoS One.
2011;6(6):e20746. doi 10.1371/journal.pone.0020746

Barrey E., Saint-Auret G., Bonnamy B., Damas D., Boyer O., Gidrol X.
Pre-microRNA and mature microRNA in human mitochondria.
PLoS One. 2011;6(5):e20220. doi 10.1371/journal.pone.0020220

Bartel D.P. Metazoan microRNAs. Cell. 2018;173(1):20-51. doi
10.1016/j.cell.2018.03.006

Bian Z., Li L.-M., Tang R., Hou D.-X., Chen X., Zhang C.-Y., Zen K.
Identification of mouse liver mitochondria-associated miRNAs and
their potential biological functions. Cell Res. 2010;20(9):1076-1078.
doi 10.1038/cr.2010.119

Breiman L. Random forests. Mach. Learn. 2001;45(1):5-32. doi
10.1023/A:1010933404324

Chandradoss S.D., Schirle N.T., Szczepaniak M., MacRae I.J., Joo C.
A dynamic search process underlies microRNA targeting. Cell.
2015;162(1):96-107. doi 10.1016/j.cell.2015.06.032

Chen J., Lin J., Hu Y., Ye M., Yao L., Wu L., Zhang W., Wang M.,
Deng T., Guo F., Huang Y., Zhu B., Wang D. RNADisease v4.0:
an updated resource of RNA-associated diseases, providing RNAdisease
analysis, enrichment and prediction. Nucleic Acids Res.
2022;51(D1):D1397-D1404. doi 10.1093/nar/gkac814

Das S., Ferlito M., Kent O.A., Fox-Talbot K., Wang R., Liu D.,
Raghavachari N., Yang Y., Wheelan S.J., Murphy E., Steenbergen C.
Nuclear miRNA regulates the mitochondrial genome in the heart.
Circ. Res. 2012;110(12):1596-1603. doi 10.1161/CIRCRESAHA.
112.267732

Dasgupta N., Peng Y., Tan Z., Ciraolo G., Wang D., Li R. miRNAs
in mtDNA-less cell mitochondria. Cell Death Discov. 2015;1(1):
15004. doi 10.1038/cddiscovery.2015.4

Erturk E., Enes Onur O., Akgun O., Tuna G., Yildiz Y., Ari F. Mitochondrial
miRNAs (mitomiRs): their potential roles in breast and
other cancers. Mitochondrion. 2022;66:74-81. doi 10.1016/j.mito.
2022.08.002

Fan S., Tian T., Chen W., Lv X., Lei X., Zhang H., Sun S., Cai L.,
Pan G., He L., Ou Z., Lin X., Wang X., Perez M.F., Tu Z., Ferrone
S., Tannous B.A., Li J. Mitochondrial miRNA determines
chemoresistance by reprogramming metabolism and regulating mitochondrial
transcription. Cancer Res. 2019;79(6):1069-1084. doi
10.1158/0008-5472.CAN-18-2505

Gohel D., Singh R. Different platforms for mitomiRs in mitochondria:
emerging facets in regulation of mitochondrial functions. Mitochondrion.
2022;66:67-73. doi 10.1016/j.mito.2022.08.003

Grimson A., Srivastava M., Fahey B., Woodcroft B.J., Chiang H.R.,
King N., Degnan B.M., Rokhsar D.S., Bartel D.P. Early origins and
evolution of microRNAs and Piwi-interacting RNAs in animals.
Nature.
2008;455(7217):1193-1197. doi 10.1038/nature07415

Grosswendt S., Filipchyk A., Manzano M., Klironomos F., Schilling
M., Herzog M., Gottwein E., Rajewsky N. Unambiguous identification
of miRNA: target site interactions by different types of
ligation reactions. Mol. Cell. 2014;54(6):1042-1054. doi 10.1016/
j.molcel.2014.03.049

Huang H.-Y., Lin Y.-C.-D., Li J., Huang K.-Y., Shrestha S., Hong H.- C.,
Tang Y., Chen Y.-G., Jin C.-N., Yu Y., Xu J.-T., Li Y.- M., Cai X.- X.,
Zhou Z.-Y., Chen X.-H., Pei Y.-Y., Hu L., Su J.- J., Cui S.-D.,
Wang F., Xie Y.-Y., Ding S.-Y., Luo M.-F., Chou C.-H., Chang N.- W.,
Chen K.-W., Cheng Y.-H., Wan X.-H., Hsu W.-L., Lee T.-Y.,
Wei F.- X., Huang H.-D. miRTarBase 2020: updates to the experimentally
validated microRNA-target interaction database. Nucleic
Acids Res. 2020;48(D1):D148-D154. doi 10.1093/nar/gkz896

Jagannathan R., Thapa D., Nichols C.E., Shepherd D.L., Stricker J.C.,
Croston T.L., Baseler W.A., Lewis S.E., Martinez I., Hollander J.M.
Translational regulation of the mitochondrial genome following
redistribution of mitochondrial microRNA in the diabetic heart.
Circ. Cardiovasc. Genet. 2015;8(6):785-802. doi 10.1161/CIRC
GENETICS.115.001067

Kanehisa M., Furumichi M., Tanabe M., Sato Y., Morishima K. KEGG:
new perspectives on genomes, pathways, diseases and drugs. Nucleic
Acids Res. 2017;45(D1):D353-D361. doi 10.1093/nar/gkw1092

Khorsandi S.E., Salehi S., Cortes M., Vilca-Melendez H., Menon K.,
Srinivasan P., Prachalias A., Jassem W., Heaton N. An in silico argument
for mitochondrial microRNA as a determinant of primary
non function in liver transplantation. Sci. Rep. 2018;8(1):3105. doi
10.1038/s41598-018-21091-9

Kozomara A., Birgaoanu M., Griffiths-Jones S. miRBase: from microRNA
sequences to function. Nucleic Acids Res. 2019;47(D1):
D155-D162. doi 10.1093/nar/gky1141

Kren B.T., Wong P.Y.-P., Sarver A., Zhang X., Zeng Y., Steer C.J.
MicroRNAs identified in highly purified liver-derived mitochondria
may play a role in apoptosis. RNA Biol. 2009;6(1):65-72. doi
10.4161/rna.6.1.7534

Li P., Jiao J., Gao G., Prabhakar B.S. Control of mitochondrial activity
by miRNAs. J. Cell. Biochem. 2012;113(4):1104-1110. doi 10.1002/
jcb.24004

Lin H.-Y., Chu P.-Y. Advances in understanding mitochondrial microRNAs
(mitomiRs) on the pathogenesis of triple-negative breast
cancer (TNBC). Oxid. Med. Cell. Longev. 2021;2021:5517777. doi
10.1155/2021/5517777

Lung B., Zemann A., Madej M.J., Schuelke M., Techritz S., Ruf S.,
Bock R., Hüttenhofer A. Identification of small non-coding RNAs
from mitochondria and chloroplasts. Nucleic Acids Res. 2006;
34(14):3842-3852. doi 10.1093/nar/gkl448

Mercer T.R., Neph S., Dinger M.E., Crawford J., Smith M.A., Shearwood
A.-M.J., Haugen E., Bracken C.P., Rackham O., Stamatoyannopoulos
J.A., Filipovska A., Mattick J.S. The human mitochondrial
transcriptome. Cell. 2011;146(4):645-658. doi 10.1016/j.cell.2011.
06.051

Mustafin Z.S., Zamyatin V.I., Konstantinov D.K., Doroshkov A.V.,
Lashin S.A., Afonnikov D.A. Phylostratigraphic analysis shows the
earliest origination of the abiotic stress associated genes in A. thaliana.
Genes. 2019;10(12):963. doi 10.3390/genes10120963

Pozniak T., Shcharbin D., Bryszewska M. Circulating microRNAs in
medicine. Int. J. Mol. Sci. 2022;23(7):3996. doi 10.3390/ijms2307
3996

Russo F., Di Bella S., Vannini F., Berti G., Scoyni F., Cook H.V., Santos
A., Nigita G., Bonnici V., Laganà A., Geraci F., Pulvirenti A.,
Giugno R., De Masi F., Belling K., Jensen L.J., Brunak S., Pellegrini
M., Ferro A. miRandola 2017: a curated knowledge base of noninvasive
biomarkers. Nucleic Acids Res. 2018;46(D1):D354-D359.
doi 10.1093/nar/gkx854

Salim U., Kumar A., Kulshreshtha R., Vivekanandan P. Biogenesis,
characterization, and functions of mirtrons. WIREs RNA. 2022;
13(1):e1680. doi 10.1002/wrna.1680

Salomon W.E., Jolly S.M., Moore M.J., Zamore P.D., Serebrov V.
Single-
molecule imaging reveals that Argonaute reshapes the binding
properties of its nucleic acid guides. Cell. 2015;162(1):84-95.
doi 10.1016/j.cell.2015.06.029

Sayers E.W., Bolton E.E., Brister J.R., Canese K., Chan J., Comeau
D.C., Connor R., Funk K., Kelly C., Kim S., Madej T., Marchler-
Bauer A., Lanczycki C., Lathrop S., Lu Z., Thibaud-Nissen F.,
Murphy T., Phan L., Skripchenko Y., Tse T., Wang J., Williams R.,
Trawick B.W., Pruitt K.D., Sherry S.T. Database resources of the
national center for biotechnology information. Nucleic Acids Res.
2022;50(D1):D20-D26. doi 10.1093/nar/gkab1112

Schriml L.M., Munro J.B., Schor M., Olley D., McCracken C., Felix V.,
Baron J.A., Jackson R., Bello S.M., Bearer C., Lichenstein R., Bisordi
K., Dialo N.C., Giglio M., Greene C. The Human Disease
Ontology 2022 update. Nucleic Acids Res. 2022;50(D1):D1255-
D1261. doi 10.1093/nar/gkab1063

Sherry S.T. dbSNP: the NCBI database of genetic variation. Nucleic
Acids Res. 2001;29(1):308-311. doi 10.1093/nar/29.1.308

Sripada L., Tomar D., Prajapati P., Singh Rochika, Singh A.K., Singh
Rajesh. Systematic analysis of small RNAs associated with human
mitochondria by deep sequencing: detailed analysis of mitochondrial
associated miRNA. PLoS One. 2012;7(9):e44873. doi 10.1371/
journal.pone.0044873

Tastsoglou S., Miliotis M., Kavakiotis I., Alexiou A., Gkotsi E.C., Lambropoulou
A., Lygnos V., Kotsira V., Maroulis V., Zisis D., Skoufos
G., Hatzigeorgiou A.G. PlasmiR: a manual collection of circulating
microRNAs of prognostic and diagnostic value. Cancers. 2021;
13(15):3680. doi 10.3390/cancers13153680

Tomasetti M., Santarelli L., Neuzil J., Dong L. MicroRNA regulation
of cancer metabolism: role in tumour suppression. Mitochondrion.
2014;19:29-38. doi 10.1016/j.mito.2014.06.004

Wang W.-X., Visavadiya N.P., Pandya J.D., Nelson P.T., Sullivan P.G.,
Springer J.E. Mitochondria-associated microRNAs in rat hippocampus
following traumatic brain injury. Exp. Neurol. 2015;265:84-93.
doi 10.1016/j.expneurol.2014.12.018

Wang X., Song C., Zhou X., Han X., Li J., Wang Z., Shang H., Liu Y.,
Cao H. Mitochondria associated microRNA expression profiling of
heart failure. BioMed Res. Int. 2017;2017:4042509. doi 10.1155/
2017/4042509

Zhang X., Zuo X., Yang B., Li Z., Xue Y., Zhou Y., Huang J., Zhao X.,
Zhou J., Yan Y., Zhang H., Guo P., Sun H., Guo L., Zhang Y.,
Fu X.- D. MicroRNA directly enhances mitochondrial translation
during muscle differentiation. Cell. 2014;158(3):607-619. doi
10.1016/j.cell.2014.05.047

Zheng H., Liu J., Yu J., McAlinden A. Expression profiling of mitochondria-
associated microRNAs during osteogenic differentiation
of human MSCs. Bone. 2021;151:116058. doi 10.1016/j.bone.2021.
116058

Ziętara K.J., Lejman J., Wojciechowska K., Lejman M. The importance
of selected dysregulated microRNAs in diagnosis and prognosis of
childhood B-cell precursor acute lymphoblastic leukemia. Cancers.
2023;15(2):428. doi 10.3390/cancers15020428

